# Relationship between maternal and/or newborn cholesterol levels and neonatal septicemia: protocol for a Ugandan cohort of mother-newborn pairs

**DOI:** 10.1186/s12887-022-03494-w

**Published:** 2022-07-20

**Authors:** Kenneth Ssebambulidde, Anthony Kayiira, Ivan Segawa, Sylvia Namanda, Victoria Nakibuuka, Victor Musiime, Theresa H. Ward

**Affiliations:** 1 Research Department, Infectious Diseases Institute, Makerere University, P.O. Box 22418, Kampala, Uganda; 2 Department of Obstetrics and Gynecology, St Francis Hospital Nsambya, Kampala, Uganda; 3 College of Health Sciences, Makerere University, Kampala, Uganda; 4 Department of Pediatrics, St Francis Hospital Nsambya, Kampala, Uganda; 5 Department of Pediatrics, College of Health Sciences, Makerere University, Kampala, Uganda; 6 Research Department, Joint Clinical Research Centre, Kampala, Uganda; 7 Department of Infection Biology, Faculty of Infectious and Tropical Diseases, London School of Hygiene and Tropical Medicine, London, UK

**Keywords:** Neonatal Septicemia, Cholesterol, Maternal Cholesterol, Newborn, Cord blood

## Abstract

**Background:**

Many aspects of microbial dissemination appear to vary with host cholesterol levels. Since neonatal septicemia remains a leading cause of newborn admissions and mortality in resource-limited settings, the contribution of abnormal cholesterol levels in maternal and/or newborn blood to the risk of neonatal septicemia and outcome requires elucidation. We aim to determine a relationship between maternal serum and neonatal cord blood cholesterol levels and neonatal septicemia.

**Methods:**

This will be a mother-newborn pair cohort study. Approximately 353 pregnant women who are eligible and consent to participate in the study will have blood drawn for a lipid profile. Upon delivery, we will analyse the cord blood cholesterol of their newborns and follow them for 28 days to determine whether the infants develop clinical signs and symptoms suggestive of neonatal septicemia. Relative risk will be used to determine the association between cholesterol and newborn septicemia. Poisson regression will be used to estimate the relative risk (with 95% confidence intervals) of developing septicemia.

**Discussion:**

Findings from our study will contribute evidence to support the inclusion of lipid profile screening for pregnant women and newborns. Our study will determine whether newborns with abnormal cholesterol or those born to mothers with abnormal cholesterol will require rigorous follow-up in neonatal clinics.

## Background

Neonatal septicemia is a leading cause of under-five mortality and morbidity. Globally, of the estimated 6.2 million children under 15 years who died in 2018, about half (2.5 million) occurred in children less than 28 days of life [1]. Moreover, neonatal septicemia was the leading cause of death in children less than 28 days of life [1]. There is a disproportionate distribution of these infections and deaths, most of which occur in sub-Saharan Africa and Southeast Asia. There are 380,000 to 2,000,000 estimated annual cases of neonatal septicemia and 270,000 associated deaths in sub-Saharan Africa [1, 2]. This translates to an economic burden of over $10 billion to $469 billion [2]. Septicemia is diagnosed in 44.9% of hospitalized neonates in Uganda, with 26.9% mortality [3]. Prolonged labor, premature rupture of membranes, prematurity, and maternal infections are among the leading risk factors for neonatal septicemia [4, 5].

Changes in lipid profiles have been associated with sepsis in different settings. After evaluating 30,239 adult individuals, Guirgis et al*.* (2016) found that low serum levels of low-density protein cholesterol (LDL-C) were associated with higher admission rates for sepsis [6]. Additionally, they found that high-density lipoprotein cholesterol (HDL-C) serum levels were not associated with sepsis events. Kaysen et al*.* (2018) reported that higher levels of LDL-C and HDL-C were associated with a lower risk of death of an infectious cause [7]. Earlier work from Iribarren et al*.* (1998) showed an inverse association between total serum cholesterol and incidence of infections [8]. These findings demonstrate a relationship between cholesterol levels, infection rates, and mortality. However, as all these studies were conducted among adults in resource-rich settings, the need to understand any link to infant outcome remains.

Although the exact role of cholesterol in the aetiopathogenesis of infections is unknown, in vitro studies have shown that immune cells require optimal membrane cholesterol for phagocytosis of pathogens. For example, phagocytosis of *Pseudomonas aeruginosa* by dendritic cells and *Cryptococcus neoformans* by macrophages requires membrane cholesterol [9, 10]. Additionally, findings from our in vitro experiments have shown that internalization of *Escherichia coli* K1 by macrophages requires membrane cholesterol, as demonstrated by a reduction in the concentration of intracellular bacteria with acute depletion of the macrophage membrane cholesterol [11]. The hematogenous dissemination of *Escherichia coli* K1 into the brain via human brain endothelial cells requires membrane cholesterol in these endothelial cells [12].

Neonates have both endogenous and exogenous sources of cholesterol [13]. The exogenous source depends on maternal cholesterol levels that increase with weeks of gestation [14–16]. Total cholesterol in the neonatal period differs by gestation age with preterm neonates having higher cholesterol levels than term neonates [17, 18]. Furthermore, literature on the association between maternal cholesterol and neonatal septicemia is meagre. Earlier studies that elucidated the role of cholesterol in sepsis were done among adults from resource-rich settings[6–8]. In this study, we seek to determine the relationship between maternal and newborn cholesterol levels and the association between maternal/neonatal cholesterol and neonatal septicemia.

## Methods

### Study design and setting

**Fig. 1:**
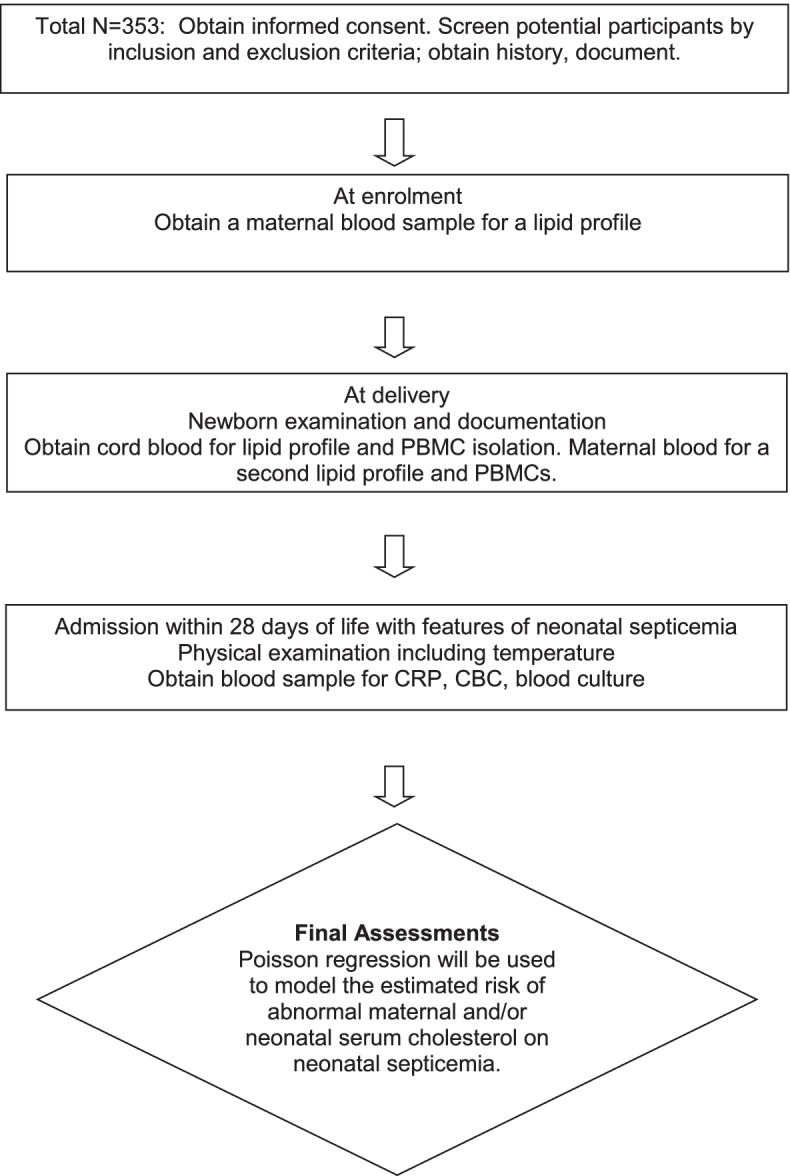
Schematic of Study Design

This will be a prospective cohort study enrolling pregnant women and their newborns. The exposure group will include pregnant women and neonates with abnormal cholesterol levels and the non-exposure group will have normal cholesterol levels. The study will be conducted at St. Francis Hospital, Nsambya in Kampala, Uganda. St. Francis Hospital is a private-not-for-profit hospital in Uganda with over 19,000 admissions and 5,500 deliveries annually run by the Kampala Catholic archdiocese. Recruitment began on 17 November 2021. The newborns will be followed up for 28 days after birth to determine whether they develop septicemia (Fig. 1).

### Participants

Pregnant women will be included in this study if they are 18 years and older, at 36 weeks of gestation or more, presenting at St. Francis Hospital Nsambya during the study period, willing to complete study procedures, and provide informed consent for themselves and their newborns. Pregnant women who present to the hospital as referrals and newborns with birth asphyxia and congenital abnormalities will be excluded.

### Variables

Our primary outcome is neonatal septicemia, defined as an infection within the first 28 days of life manifesting with the following: (1) symptoms such as lethargy or altered mentation, refusal to feed, irritability, difficulty in breathing, vomiting, or fever, (2) a temperature above 37.5 °C or below 35 °C, heart rate > 180 beats/min or < 100 beats/min, respiratory rate > 60 breaths/min plus grunting or desaturation, (3) raised serum C Reactive Protein > 10 mg/mL, leucocytosis (WBC > 34,000 × 10^9^)/leukopenia (WBC < 5000 × 10^9^), thrombocytopenia < 100,000 × 10^9^ with or without a positive blood culture.

Our secondary outcomes include: (1) correlation between maternal serum cholesterol levels and cord blood neonatal cholesterol levels at birth, and (2) correlation between maternal serum and cord blood cholesterol with maternal and newborn peripheral blood mononuclear cell membrane cholesterol levels at birth. The exposures are maternal and newborn cord blood total cholesterol level at delivery. Abnormal maternal total serum cholesterol is either below 219 mg/dL (5.67 mmol/L) or above 349 mg/dl (9.04 mmol/L) [19]. Abnormal newborn cord blood total cholesterol level at delivery will be defined as less than 43 mg/dL or higher than 93 mg/dL [20].

### Study procedures

**Fig. 2:**
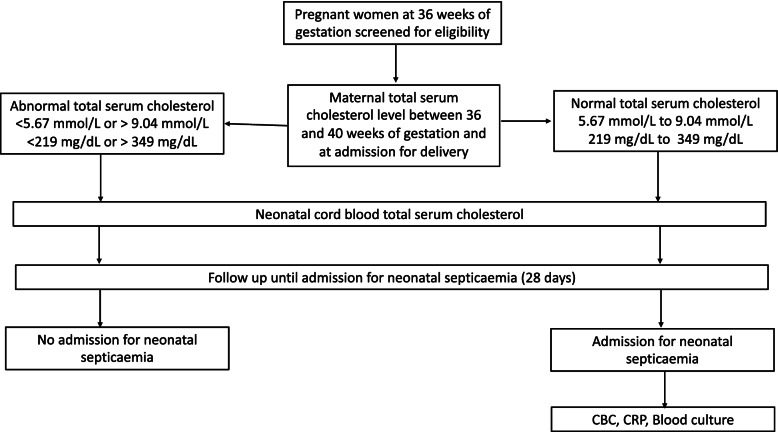
Study flow

Pregnant women attending antenatal care at Nsambya Hospital will be approached and provided information about the study (Fig. 2). At 36 weeks of gestation, eligible pregnant women who consent will be assigned a study number. We will obtain socio-demographic and pertinent clinical data. Venous blood will be drawn for a lipid profile analysis, including total cholesterol, low-density lipoprotein, high-density lipoprotein, and triglycerides [first maternal serum lipid profile]. On presenting to the labor ward, a second maternal serum lipid profile will be done, and a blood sample for peripheral blood mononuclear cells (PBMCs) isolation will be collected (Table 1). We will collect the birth order, gestation age at delivery, duration of labor, mode of delivery, prior history of maternal infection during the pregnancy resulting in the neonate’s delivery, maternal history of chronic illnesses like Diabetes mellitus, asthma, or hypertension, maternal age, maternal level of education and employment status. Pregnant women who eventually fail to deliver at Nsambya hospital will be replaced a posteriori.

**Table 1:** Study procedures

Protocol activity	Screening	Enrolment	At labor ward admission	Post Delivery	Admission for neonatal sepsis
Assessment for eligibility	X	X			
Informed consent		X			
Maternal medical history	X	X	X		
Maternal lipid profile		X	X		
Newborn’s lipid profile on cord blood and PBMC isolation				X	
Newborn assessment- birth weight, length, abdominal circumference				X	X
CBC					X
CRP					X
Blood culture					X

*CBC* complete blood count, *CRP* C-reactive protein, *PBMC* peripheral blood mononuclear cells

After delivery, cord blood will be collected for a lipid profile, and another sample of cord blood will be collected for PBMCs isolation. The isolated maternal and neonate PBMCs will be stored at -80˚C for PBMC membrane cholesterol quantification by flow cytometry and future studies. Newborns will undergo newborn assessment, including birth weight, sex, length, head circumference, and abdominal circumference. Newborns will be followed up weekly for 28 days post-delivery.

Newborns who develop signs and symptoms of neonatal septicemia will be admitted for evaluation and management. These signs and symptoms include fever, irritability, refusal to feed, vomiting, fast breathing, or seizures. These neonates will then have blood drawn for C-reactive protein, complete blood count and blood culture. They will receive treatment as per established guidelines in the hospital. Their treatment, duration of hospitalization, and treatment outcome will be collected. The status at 28 days of life of all enrolled newborns will be collected.

All samples will be drawn in specimen tubes pre-labelled with the study participants’ study identification number unique to each mother-baby pair. These samples will be stored in a cooler box before being transported either to the Nsambya hospital laboratory, where lipid profiles will be done, and or to the Infectious Diseases Institute (IDI) translational laboratory for PBMC isolation. Following their isolation, PBMCs will be stored at -80˚C in 4 aliquots of 1 ml each at the IDI.

## Sample size

Previous studies reported the proportion of newborns in Uganda that were admitted with neonatal sepsis (clinical and culture proven) to range from 0.11 to 0.49 [21, 22]. Considering budgetary restrictions in a proof-of-concept study, we wished to determine the minimum detectable change in effect size (relative risk) for a range of sample sizes restricted between 100 to 500 with 80% power using a two-sided 5% level test. For sample size estimation, we used STATA version 16 (StataCorp, College Station, TX, USA), power analysis using Pearson X2 statistics for two independent proportions. We assumed the proportion in control group (normal cholesterol) to be 0.11 (lower limit of the general population), measure of effect as relative risk, and higher proportion in the exposure group (abnormal cholesterol). We estimated experimental-group proportions for a two sample proportions using Pearson's chi-squared test. A total sample size of 300 mother-newborn pairs will enable us to detect a 2.1 relative risk for neonatal septicemia in newborns of mothers with abnormal total serum cholesterol level, with 80% power and type I error of 5%. A final sample size of 353 mother-newborn pairs was determined after adjusting for 15% loss to follow up.

## Analysis of endpoints

Data will be analyzed using STATA 16. Numerical variables such as age will be summarized using means (with standard deviations) or medians (with interquartile ranges) as appropriate. Categorical variables will be summarized using proportions. Participant demographics and baseline characteristics will be grouped into respective exposure (abnormal total serum cholesterol levels (high or low)) and non-exposure groups (total serum cholesterol within the normal range). The student t-test or Wilcoxon rank-sum test will be used to compare numerical variables across the exposure groups as appropriate. A chi-square statistic (or Fischer’s exact test) will be used to determine any differences in proportions between the exposure groups. Poisson regression will be used to model the estimated risk of abnormal maternal and/or neonatal serum cholesterol on neonatal admission for septicemia. The models will be adjusted for hypothesized confounding variables and the estimates presented as crude and adjusted relative risk with their 95% confidence intervals. Maternal-related confounding variables will include age, body mass index, socio-economic status, level of education, family history of cholesterol disorders, partner level of education and socio-economic status. Newborn-related confounding variables will include gestation age at birth, sex, and birth weight. On top of these a priori defined confounders, we shall also adjust for confounders identified while building the final model of outcome and predictors. Confounders will be those factors that alter the estimate of measure of association by more than 10%.

## Discussion

This prospective cohort study will investigate the association between maternal third trimester and/or newborn cholesterol and neonatal septicemia. Findings are likely to guide routine utilization of lipid profile assessment during antenatal care to stratify newborns born to mothers with abnormal cholesterol levels who will need more regular follow-up and prompt recognition and management of neonatal septicemia.

Maternal total cholesterol levels increase during pregnancy. Maternal total cholesterol is associated with birth weight in both resource-rich and resource-limited settings [23–25]. Whether mothers with higher or lower total cholesterol expected for gestation weeks give birth to newborns with higher relative risk for poor newborn outcomes in resource-limited settings remains to be evaluated. Larger evaluation of adding a lipid profile to the antenatal care package in resource-limited settings to predict neonatal infections may be recommended basing on findings from this cohort study.

The study will look at both serum lipid profile and quantification of membrane cholesterol in peripheral blood mononuclear cells isolated from both maternal blood and cord blood at birth. Whether serum or cord blood cholesterol levels correlate with immune cell (PBMCs) membrane cholesterol will be evaluated to add weight to lipid profiling.

## Data Availability

Maternal and newborn PBMCs isolated during the study will be stored frozen (at -80 °C or liquid nitrogen) for future studies. Storage will be at the IDI for the first two years of the study and thereafter will either be shifted to the Makerere University biorepository or the LSHTM/UVRI/MRC biobank in Entebbe when funds are available for this in-country longer term storage. In case of unavailability of funds for longer term storage in Uganda, samples will be shipped to the LSHTM in London for longer term storage pending future studies. Consent will be obtained for long term storage with an option for participants to either agree, or not, for storage to enable future studies into further immunological or biochemical factors. De-identified data will be made publicly available on Mendeley Data (https://data.mendeley.com/). Data ownership will be in accordance with the approved data sharing agreement.

## Abbreviations

CBC: Complete blood count
CRP: C-reactive protein
HDL-C: High Density Lipoprotein Cholesterol
IDI: Infectious Diseases Institute
IRB: Institutional Review Board
LDL-C: Low Density Lipoprotein Cholesterol
LTFU: Loss to follow up
PBMCs: Peripheral blood mononuclear cells
TC: Total Cholesterol
